# Proteomic Analysis of Liver from Finishing Beef Cattle Supplemented with a Rumen-Protected B-Vitamin Blend and Hydroxy Trace Minerals

**DOI:** 10.3390/ani11071934

**Published:** 2021-06-29

**Authors:** Mariana Mescouto Lopes, Thaís Ribeiro Brito, Josiane Fonseca Lage, Thaís Correia Costa, Marta Maria dos Santos Fontes, Nick Vergara Lopes Serão, Tiago Antônio de Oliveira Mendes, Ricardo Andrade Reis, Renata Veroneze, Fabyano Fonseca e Silva, Marcio de Souza Duarte

**Affiliations:** 1 Department of Animal Science, Universidade Federal de Viçosa, Viçosa 36570-000, Brazil; mariana.mescouto@ufv.br (M.M.L.); thais.correia@ufv.br (T.C.C.); marta.fontes@ufv.br (M.M.d.S.F.); renata.veroneze@ufv.br (R.V.); fabyanofonseca@ufv.br (F.F.e.S.); 2 Muscle Biology and Nutrigenomics Laboratory, Universidade Federal de Viçosa, Viçosa 36570-000, Brazil; 3 Department of Animal Science, São Paulo State University, Jaboticabal 14887-900, Brazil; tr.brito@unesp.br (T.R.B.); rareis@fcav.unesp.br (R.A.R.); 4 Trouw Nutrition, Research and Development, Campinas 13080-650, Brazil; josiane.lage@trouwnutrition.com; 5 Department of Animal Science, Iowa State University, Ames, IA 50011, USA; serao@iastate.edu; 6 Department of Biochemistry and Molecular Biology, Universidade Federal de Viçosa, Viçosa 36570-000, Brazil; tiagoaomendes@ufv.br

**Keywords:** beef cattle, proteomics, vitamin B, hydroxyl trace minerals, liver metabolism

## Abstract

**Simple Summary:**

Greater metabolic needs in high-producing beef cattle might lead to mineral and vitamin deficiency. Previous studies have shown the benefits of B-vitamin and trace mineral supplementation in animal performance of ruminants; however, little is known about the effects of supplementing finishing beef cattle with rumen-protected forms on the liver metabolism. Therefore, the aim of the present study was to determinate the impact of rumen-protected B-vitamin blend and hydroxy trace mineral supplementation on the hepatic proteome of finishing steers. This study reports the first evidence indicating that the supplementation of these micronutrients induces protein changes concerning oxidative metabolism and responses to oxidative stress in the liver tissue.

**Abstract:**

Vitamin B and trace minerals are crucial molecular signals involved in many biological pathways; however, their bioavailability is compromised in high-producing ruminant animals. So far, studies have mainly focused on the effects of these micronutrients on animal performance, but their use in a rumen-protected form and their impact on liver metabolism in finishing beef cattle is poorly known. We used a shotgun proteomic approach combined with biological network analyses to assess the effects of a rumen-protected B-vitamin blend, as well as those of hydroxy trace minerals, on the hepatic proteome. A total of 20 non-castrated Nellore males with 353 ± 43 kg of initial body weight were randomly assigned to one of the following treatments: CTRL—inorganic trace minerals without supplementation of a protected vitamin B blend, or SUP—supplementation of hydroxy trace minerals and a protected vitamin B blend. All animals were fed the same amount of the experimental diet for 106 days, and liver biopsies were performed at the end of the experimental period. Supplemented animals showed 37 up-regulated proteins (*p* < 0.10), and the enrichment analysis revealed that these proteins were involved in protein folding (*p* = 0.04), mitochondrial respiratory chain complex I (*p* = 0.01) and IV (*p* = 0.01), chaperonin-containing T-complex 2 (*p* = 0.01), glutathione metabolism (*p* < 0.01), and other aspects linked to oxidative-stress responses. These results indicate that rumen-protected vitamin B and hydroxy trace mineral supplementation during the finishing phase alters the abundance of proteins associated with the electron transport chain and other oxidation–reduction pathways, boosting the production of reactive oxygen species, which appear to modulate proteins linked to oxidative-damage responses to maintain cellular homeostasis.

## 1. Introduction

Micronutrients, like vitamins, carotenoids, and minerals, act as signals that regulate gene expression and, subsequently, the mRNA, protein, and metabolite levels [1]. Consequently, the lack of these bioactive compounds may lead to alteration of DNA methylation patterns, impacting gene expression and protein abundance [2]. In this sense, complex vitamin B molecules play a vital role in the activation of several enzymes that constitute biological pathways of carbohydrate, lipid, protein, and one-carbon metabolism, such as methionine and folate cycles, besides contributing to antioxidant defense [3]. B vitamins are water-soluble, hence are not stored, and are synthesized by ruminal bacteria, leading to the belief that they are unnecessary to supplement [4]. However, as a consequence of their consumtion at greater concentrations, physiological changes are observed in the ruminal environment, which may compromise the absorption and metabolism of certain nutrients. In this scenario, several studies have shown the benefits of B-vitamin supplementation in attending to the requirements of high-producing animals, especially when provided in a rumen-protected form [5,6,7,8]. As such, to increase the intake of these vitamins in the intestine, encapsulation technology in a lipid matrix might be employed. 

Besides vitamins, trace minerals (TM) are fundamental nutritional compounds that participate in most biochemical reactions in the body, performing a significant role in the development and health of domestic animals [9]. TM has multiple roles as a component of metalloenzymatic complexes, controlling gene expression, appetite, fat metabolism, and immune responses (zinc), and contributes to hemoglobin formation, growth, and antioxidant defense (copper) [10,11]. The establishment of mineral requirements in livestock is a challenging procedure, since several aspects of the ingredients of the diets, such as the mineral chemical form, may affect their use by the animals [12]. The reactivity of ions from inorganic minerals leads to higher dissociation in the rumen, consequently impairing its bioavailability [13]. Less reactive sources, like hydroxy minerals, have been shown to reduce ruminal TM dissociation, which diminishes the interaction with other nutritional components, improving the digestibility of vitamins, lipids, and enzymes [14,15,16]. 

Despite the current knowledge of B-vitamin complexes’ and trace minerals’ effects on animal performance, the impact of supplementing calves at high growth rates with protected B vitamins and hydroxy sourced TM on hepatic metabolism is poorly known. The liver is a complex organ, fundamental for all metabolic processes, such as energy distribution, hunger or satiety signals integration, and nutrient processing [17,18], ultimately modulating animal performance. Thus, in the current study, we investigated the effects of hydroxy trace minerals and a rumen-protected B-vitamin blend on the hepatic proteome of beef cattle at the finishing phase on pasture.

## 2. Materials and Methods

All the protocols related to animal management and handling were approved by the Animal Care and Use Committee of the College of Agricultural and Veterinary Sciences at the Universidade Estadual Paulista ”Júlio de Mesquita Filho”, Jaboticabal, São Paulo, Brazil (protocol number 006000/19). The typical climate of the region is a subtropical humid type, with dry winters and wet summers. The pastures used were planted with *Brachiaria brizantha* (Hochst ex A. Rich) Stapf Marandu (Marandu grass).

### 2.1. Animals and Experimental Diets

Twenty non-castrated Nellore males with 353 ± 43 kg (mean ± SD) of initial body weight (BW) at the finishing phase were used. The experimental period was carried out from June to October 2019, with 14 days of adaptation to the dietary treatments, followed by 106 days of implementation of experimental treatments, totaling 120 days. 

Bulls were randomly allocated in 8 paddocks in the pasture, where they were divided into four paddocks with three animals and another four with two animals. Thus, experimental treatments were the following: Control—inorganic trace mineral without supplementation of a rumen-protected B-vitamin blend (CTRL, *n* = 11); Supplemented—with supplementation of a rumen-protected B-vitamin blend, containing pantothenic acid (B5), pyridoxine (B6), folic acid (B9), biotin (B7), and cyanocobalamin (B12) (Vivalto^®^—Trouw Nutrition, Isola Vicentina, Italy), and hydroxyl trace minerals, copper and zinc (IntelliBond^®^—Micronutrients Inc., Indianapolis, IN, USA) (SUP, *n* = 9).

Animals of both experimental groups received the same amount of concentrate (1.75% of BW/animal), which contained the same composition (15.55% of total digestible nutrients (TDN), 16.15% of crude protein, and 84.45% TDN of dry matter basis). The chemical composition of the mineral/vitamin mixture is shown in Table 1. The concentrate dry matter intake was evaluated according to the balance between food supply and leftovers of each paddock. 

### 2.2. Liver Biopsy

At the end of the finishing period, all animals were subjected to a liver biopsy. The sampling was performed via needle biopsy (Tru-Cut biopsy needle; Care Fusion Corporation, San Diego, CA, USA), according to the procedure described by Mølgaard [19]. After local anesthesia with 3 to 5 mL of lidocaine, an incision between the 11th and 12th ribs was made, and tissue samples from the right hepatic lobe were collected. Liver samples (~30 mg of tissue) were placed in cryotubes, immediately snap-frozen, and stored in liquid nitrogen until processing.

### 2.3. Protein Extraction 

Liver samples (30 mg) were homogenized using turrax (IKA ULTRA-TURRAX T18 digital, IKA, Staufen, Germany) for 10 seconds in a lysis buffer containing 7M urea, 2M thiourea, 4% 3-((3-cholamidopropyl) dimethylammonio)-1-propanesulfonate (CHAPS) detergent, 1% *dithiothreitol* (DTT), and 10 uL protease inhibitor. The supernatant was collected after centrifugation at 10,000× *g* for 30 minutes at 4 °C. The total amount of protein was quantified by the Bradford method (Bio-Rad Laboratories, Hercules, CA, USA).

### 2.4. Protein Digestion

After quantification, 50 ug of the sample was transferred to a tube and 2.5 μL of 100 mM DTT was added. The solution was then agitated and placed in a heat block at 60 °C for 30 min. 2.5 μL of 300 mM iodoacetamide was added after the vial reached room temperature, for cysteine alkylation. This compound is sensitive to light, so after agitation in a vortex, the samples were transferred to the dark, at room temperature, for 30 minutes. 10 μL of trypsin solution (Promega) was added to ammonium bicarbonate (Ambic), stirred in a vortex, and digested at 37 °C overnight. After digestion, the samples were dried in a Savant SpeedVac^TM^ concentrator (Thermo Fisher Scientific, Waltham, MA, USA), resuspended in 50 μL of 0.1% trifluoroacetic acid (TFA) solution prepared in H_2_O milliQ and concentrated with the microcolumn ZipTip^®^ C18 (Merck Millipore, Billerica, MA, USA).

### 2.5. Mass Spectrometry of Protein Samples 

Mass spectrometry analysis was performed at the Chemistry Institute (Central Analítica) at USP (São Paulo, Brazil). Peptides were separated on a C18 reverse-phase column on a 90 min gradient, and mass spectrometry was carried out on q-ToF maxis 3G (Bruker Daltonics GmBH, Bremen, Germany) coupled with Easy NanoLC II (Thermo Scientific, Waltham, MA, USA). 

The acquired data were analyzed with MaxQuant software version 1.6.10.43 (Max Plank Institute of Biochemistry, Planegg, Germany) [20] for protein identification, searched against the *Bos taurus* database obtained from UniProt (www.uniprot.org, accessed on 19 July 2020). The following parameters were used: trypsin specificity, two missed cleavages, and methionine oxidation and acetylation at protein amino-terminals were specified as variable modifications, while carbamidomethylation of cysteine was specified as a fixed modification (Supplemental Table S1). Peptide and protein false discovery rate (FDR) was set at 1%. Label-free quantification (LFQ) was performed, and only protein ratios calculated from at least two unique peptide ratios (min LFQ ratio count = 2) were considered for calculation of the LFQ protein intensity.

### 2.6. Statistical Analysis

Prior to statistical analyses, proteins that were not detected in at least 15% of animals within each treatment were removed from the dataset. These were then subjected to normalization of the library size and subsequent transformation for analyses using a linear model. The protein abundance data were used to obtain normalizing factors using the Trimmed Mean of M-values (TMM) method from the TMM package [21] implemented in R (Vienna, Austria) [22]. Afterward, the relative abundance of the normalized data was obtained and then log_2_-transformed. The data were analyzed using the following linear mixed model:

(1) $$Y_{ijk}=\mu +T_{i}+p_{j}+b_{1}\left(iBW_{k}-\bar{iBW}\right)+e_{ijk},$$

where $Y_{ijk}$ is the log_2_-transformed normalized relative abundance of the protein being analyzed; μ is the intercept; $T_{i}$ is the fixed effect of the *i*th Treatment (CRTL or SUP); $p_{j}$ is the random effect of paddock, assuming $p_{j}\sim N(0,\sigma_{p}^{2})$; $b_{1}$ is the partial regression coefficient associated in the effect of initial body weight of the animal; $iBW_{k}$ is the initial body weight of the *k*th animal; $\bar{iBW}$ is the average initial body weight of the data; and $e_{ijk}\;$ is the residual associated with $Y_{ijk}$, assuming $e_{ijk}\sim N(0,\sigma_{e}^{2})$. Prior to final analyses, assumptions of homogeneity and normality of the residuals were met. Analyses were performed in SAS 9.4 (Statistical Analysis System Institute, Inc., Cary, NC, USA) with the GLIMMIX and UNIVARIATE procedures.

After analyses, FDR [23] was used to adjust the *p*-values (*q*-values) for the effect of treatment due to multiple tests. Significant differentially abundant proteins (DAP) were identified at *q*-value < 0.10. *q*-values were obtained in R using the qvalue function of the qvalue package [24].

### 2.7. Bioinformatics Analysis 

The protein–protein interaction, Kyoto Encyclopedia of Genes and Genomes (KEGG), and Gene Ontology (GO) pathways enrichment analyses were performed with the String software 11.0 (string-db.org), using Bos taurus data and the minimum confidence score required of 0.40 [25]. The functional categorization of GO and KEGG pathways were considered enriched at FDR-adjusted *p*-value (*P*_FDR_) < 0.05 based on Benjamini-Hochberg’s method [26]. 

## 3. Results

### 3.1. Differentially Abundant Proteins

**Figure 1:**
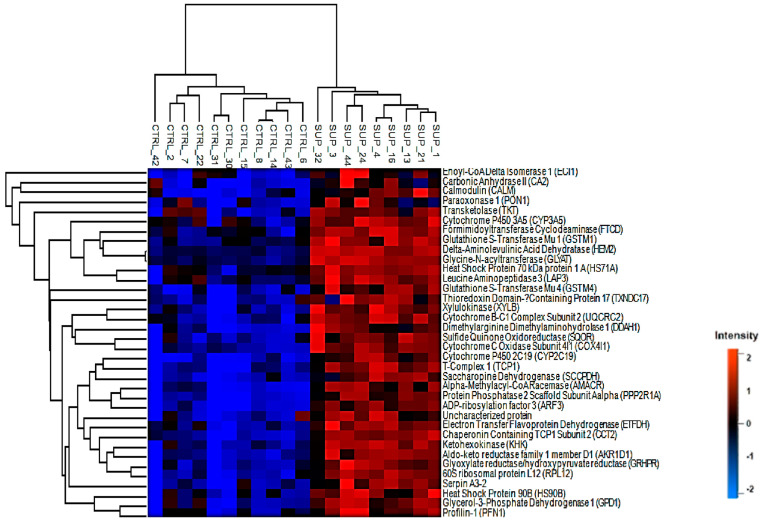
Differentially abundant proteins.

A total of 1400 groups of proteins were detected in the bovine liver samples using label-free quantitation (Supplemental Table S2), and 753 proteins were selected after filtration. There were 37 DAPs (*q*-value < 0.10 and fold change (FC) ± 2.0) between treatment groups, where all proteins were up-regulated in the SUP treatment. These results are presented in Figure 1. 

Proteins related by structural and regulatory function, such as profilin-1 (PFN1; *p =* 0.07), protein phosphatase 2 scaffold subunit beta (PPP2R1B; *p* = 0.06), protein phosphatase 2 scaffold subunit alpha (PP2R1A; *p* = 0.06), and carbonic anhydrase 2 (CA2; *p* = 0.07), are not directly involved in energy metabolism, therefore they will not be further discussed. A greater abundance of proteins involved in lipid metabolism was found in the liver of supplemented animals, for example, enoyl-CoA delta isomerase 1 (ECl1; *p* = 0.07), electron transfer flavoprotein dehydrogenase (EFDH; *p* = 0.07), and glycerol-3-phosphate dehydrogenase 1 (GPD1; *p* = 0.06). Two proteins associated with the electron transport chain, cytochrome c oxidase subunit 4I1 (COX4I1; *p* = 0.07) and ubiquinol-cytochrome c reductase core protein 2 (UQCRC2; *p* = 0.07), were also up-regulated in the supplemented group. Additionally, we detected proteins linked to detoxification and stress responses, such as cytochrome P450 family proteins (CYP3A4, *p* = 0.07, CYP2C19; *p* = 0.065, CYP3A24; *p* = 0.07), glutathione S-transferases (GSTM1; *p* = 0.01, GSTM4; *p* = 0.065), and heat-shock proteins (HSP1A; *p* = 0.07, HSP90AB1; *p* = 0.07). 

**Figure 2:**
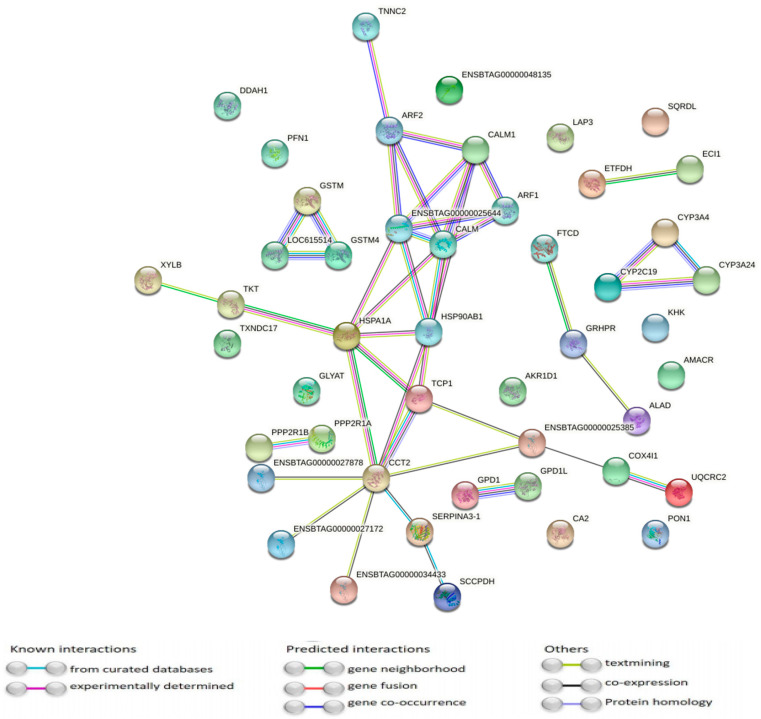
Protein interaction network of differentially abundant proteins (*q*-value < 0.10) in the liver between control and supplemented animals. Nodes represent the differentially abundant proteins, and the lines represent the connection between proteins.

The interaction network between the DAPs was highly significant (*p* < 1.43^−5^), indicating that the DAPs might be biologically relevant. Only 12 proteins did not present any interactions. These results are presented in Figure 2. 

### 3.2. Pathway Analysis of Differentially Abundant Proteins

DAPs were categorized according to GO into biological processes, cellular compartment, and molecular function. Only one enriched pathway for biological process was identified, this being related to protein folding (*P*_FDR_ = 0.04). For molecular function, the top five enriched pathways were nucleotide binding (*P*_FDR_ = 0.01), catalytic activity (*P*_FDR_ = 0.01), protein binding (*P*_FDR_ = 0.01), purine binding (*P*_FDR_ = 0.01), and protein-folding chaperones (*P*_FDR_ = 0.01). For cellular components, we found enriched cytoplasm (*P*_FDR_ = 0.002), oxidoreductase complex (*P*_FDR_ = 0.01), mitochondrial respiratory chain complex IV (*P*_FDR_ = 0.01), and chaperonin-containing T-complex 2 (*p* _FDR_ = 0.01). 

**Figure 3:**
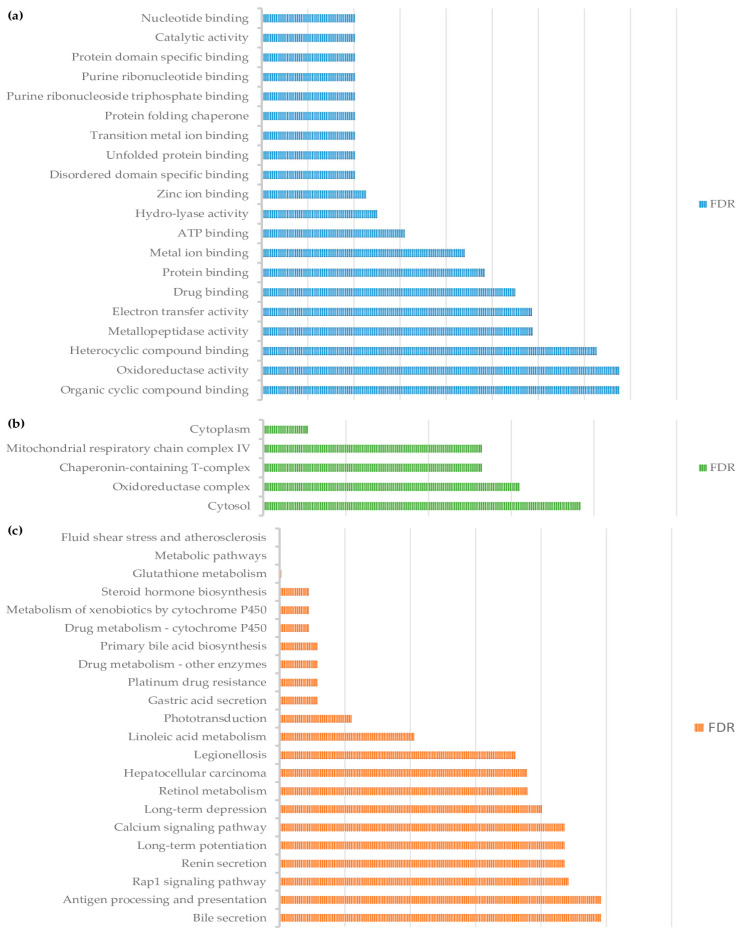
Function categorization of differentially abundant proteins: (**a**) Molecular function; (**b**) Cellular compartment; (**c**) KEGG.

The results of the KEGG pathway analysis (Figure 3) showed that supplementation of the B-vitamin blend and hydroxy trace minerals affected fluid shear stress and atherosclerosis (*P*_FDR_ < 0.01), metabolic pathways (*P*_FDR_ < 0.01), glutathione metabolism (*P*_FDR_ < 0.01), steroid hormone biosynthesis (*P*_FDR_ < 0.01), and metabolism of xenobiotics by cytochrome P450 (*P*_FDR_ < 0.01).

## 4. Discussion

The present study aimed to investigate the changes in the hepatic proteome of beef cattle at the finishing phase after vitamin B and hydroxy trace mineral supplementation. We used a shotgun proteomic approach based on LC-MS/MS to study liver samples of Nellore bulls classified according to their diets. The liver acts as a sensor of nutrient status and regulates its metabolic activity according to nutrient availability, and imbalance might lead to impaired energy supply [27]. In this sense, although the hepatic level of B vitamins, Cu, and Zn were not measured, we were able to report protein variations related to metabolic pathways up-regulated in the liver of beef cattle supplemented with a rumen-protected B-vitamin blend and hydroxy trace minerals, which suggest an effect of micronutrient bioavailability on tissue metabolism. 

Both UQCRC2 and COX4I1 are important proteins associated with complex III and IV assembly of the electron transport chain (ETC) [28], and were up-regulated in the liver of supplemented animals. In a previous study, adequate levels of vitamin B, iron, Cu, and Zn might directly have acted on complex IX activation through the heme biosynthetic pathway [29]. Copper deficiency reduces the expression and activity of complex IV, but not other complexes once it contains two molecules of heme bound with Cu centers, all of which are involved in the electron transfer process [30,31,32]. The higher mitochondrial protein content and complex protein abundance of the respiratory chain might indicate greater metabolic efficiency according to research findings in steers and broilers [33,34,35].

The up-regulation of these proteins in the liver of supplemented animals and the GO enriched pathways mitochondrial respiratory chain complex IV (GO: 0005751), electron transfer activity (GO: 0009055), and oxidoreductase complex (GO: 1990204) indicates higher activity of oxidative phosphorylation, which may be due to the oxidation of lipids, carbohydrates, and protein, to produce energy to meet energy needs for maintenance. Indeed, we found two up-regulated proteins linked to fatty acid oxidation, ECI1 and ETFDH, the latter physically interacting with ETC complex III at the coenzyme Q reduction site [36]. Our results are similar to previous studies, where the addition of copper reduced the hepatic fat content by enhancing fatty acid oxidation in rabbits [37], and rumen-protected B vitamins and choline supplementation in transition dairy cows diminished liver fat content postpartum [8]. 

Further evidence of altered lipid metabolism between treatments were the up-regulated proteins CYP2C19, CYP3A24, and CYP3A4 in the SUP group. Cytochrome P450 genes are induced by bile acids and oxysterols, coding for liver enzymes involved in major pathways of cholesterol degradation, vitamin D and bile acids metabolism, and maintaining the homeostasis of xenobiotics and other compounds of endogenous decontamination processes [38]. CYP enzymes, as well as the mitochondrial respiratory chain, are also sources of reactive oxygen species (ROS), once the normal P450 catalytic cycle generates superoxide anion (O_2_^−^) and hydrogen peroxide (H_2_O_2_) [39]. 

The accumulation of ROS causes degradation by nonspecifically attacking membranes, proteins, and nucleic acids, leading to impaired energy expenditure [40]. To diminish the oxidative stress caused by ROS, mammalian cells trigger an antioxidant defense system, which consists of antioxidant enzymes and several non-enzymatic antioxidants, such as glutathione (GSH), cysteine, thioredoxin, and vitamins [41]. Changes in GSH content may result in sub-optimal growth and altered oxidative-stress responses, since it was observed in studies with obese mice and humans that the level of GSH in skeletal muscle and adipose tissues was decreased [42,43]. The up-regulated glutathione-S-transferases (GSTM1, and GSTM4) are known for conjugating GSH to xenobiotics for detoxification [43], and their increased abundance in the liver of supplemented animals was linked to glutathione metabolism (bta00480), suggesting higher demand for GSH, and greater capacity for detoxification. However, it has been shown that the liver of finished steers, when compared to growing steers, had a larger expression of GSTM1, but did not present any change in GSH content [44]. 

Another indication of hepatic cytoprotective effects by the rumen-protected B-vitamin blend and hydroxy trace minerals in response to ROS production is the level of highly-regulated proteins named “heat-shock proteins” (HSPs). The HSPs concentration in the cell may increase in response to stress signals, such as oxidative stress, inflammatory conditions, toxic stress, and environmental challenges [45], once they are capable of inhibiting pro-inflammatory/apoptotic pathways through the modulation of nuclear factor (NF-kB) and the activation of caspases as well as the c-Jun NH2-terminal kinase pathway [46]. In accordance, HSPA1A, HSP90AB1, TCP1, and CCT2 were up-regulated in the liver of supplemented animals, indicating greater oxidative-stress status, likely due to a higher metabolic rate. Nonetheless, these proteins were associated with enriched terms related to protein-folding processes, which suggests enhanced ability of cell defense against cellular oxidative-stress toxic effects [47]. 

## 5. Conclusions

The present study provided the first evidence that protected vitamin B and hydroxy trace mineral supplementation during the finishing phase alters the hepatic proteome in beef cattle. Although we have not measured blood levels of B vitamins and trace minerals, our data suggest that higher bioavailability of B vitamins, Cu, and Zn acts directly on the abundance of proteins related to the electron transport chain and other oxidation-reduction pathways, boosting the production of reactive oxygen species. Such alterations appear to modulate proteins linked to oxidative-damage responses in the liver to maintain cellular homeostasis. More research is warranted to better examine the biological mechanism of these micronutrients on metabolic pathways. 

## Figures and Tables

**Table 1:** Chemical composition of mineral/vitamin mixture.

	Control	Supplemented	Units
Ca	140	140	g kg^−1^
P	28	28	g kg^−1^
Na	75	75	g kg^−1^
K	46	46	g kg^−1^
Mg	64	64	g kg^−1^
S	23	23	mg kg^−1^
Zn	1150 ^1^	1150 ^2^	mg kg^−1^
Cu	312 ^1^	312 ^2^	mg kg^−1^
F	465	465	mg kg^−1^
Mn	1080	1080	mg kg^−1^
Co	31	31	mg kg^−1^
I	22	22	mg kg^−1^
Vitamin A	62,310	62,310	UI kg^−1^
Vitamin D3	8830	8830	UI kg^−1^
Vitamin E	860	860	UI kg^−1^
Vitamin B6	-	161	UI kg^−1^
Vitamin B12	-	1.934	ug kg^−1^
Vitamin B3	-	20.000	mg kg^−1^
Vitamin B9	-	2.175	mg kg^−1^
Vitamin B7	-	1.615	mg kg^−1^
Monensin	600	600	mg kg^−1^

^1^ Inorganic mineral, ^2^ Hydroxy mineral.

## Data Availability

Data is contained within the article and supplementary materials.

## Supplementary Materials

The following are available online at https://www.mdpi.com/article/10.3390/ani11071934/s1, Table S1: Summary information of mass spectrometry analysis, Table S2: List of proteins identified in liver tissue of beef cattle supplemented or not with vitamin B and hydroxyl minerals during the finishing phase and their raw intensities.
